# Association of Toll-Like Receptor 4 Gene Polymorphism and Expression with Urinary Tract Infection Types in Adults

**DOI:** 10.1371/journal.pone.0014223

**Published:** 2010-12-03

**Authors:** Xiaolin Yin, Tianwen Hou, Ying Liu, Jing Chen, Zhiyan Yao, Cuiqing Ma, Lijuan Yang, Lin Wei

**Affiliations:** 1 Department of Immunology, Hebei Medical University, Shijiazhuan, China; 2 Department of Laboratory Medicine, Bethune International Peace Hospital, Shijiazhuan, China; Institut Pasteur, France

## Abstract

**Background:**

Innate immunity of which Toll-like receptor (TLR) 4 and CXCR1 are key elements plays a central role in the development of urinary tract infection (UTI). Although the relation between the genetics of TLR4 and CXCR1 and UTI is investigated partly, the polymorphisms and expression of TLR4 and CXCR1 in different types of UTI in adults are not extremely clear.

**Methodology/Principal Findings:**

This study investigates the presence of TLR4 A (896) G and CXCR1 G (2608) C polymorphisms in 129 UTI patients using RFLP-PCR. Gene and allelic prevalence were compared with 248 healthy controls. Flow cytometry was used to detect TLR4 and CXCR1 expression in the monocytes of UTI patients and healthy controls. TLR4 (896) AG genotype and TLR4 (896) G allele had higher prevalence in UTI (especially in acute cystitis and urethritis) patients, whereas CXCR1 (2608) GC genotype and CXCR1 (2608) C allele had lower prevalence in UTI patients than controls. TLR4 expression was significantly lower in chronic UTI patients than in acute pyelonephritis or healthy controls. CXCR1 expression was similar in both controls and patients. TLR4 expression in chronic UTI patients after astragalus treatment was higher than pre-treatment.

**Conclusions:**

The results indicate the relationship between the carrier status of TLR4 (896) G alleles and the development of UTI, especially acute cystitis and urethritis, in adults. TLR4 expression levels are correlated with chronic UTI.

## Introduction

Urinary tract infections (UTIs) are common infectious diseases,and the non-complex UTI cases show no obvious behavioral, functional, and structural risk factors. The early response against UTI is provided by innate immune response, in which, TLR4 plays an important role in activating innate immune response, and its recognized ligands, such as lipopolysaccharide, have been demonstrated to initiate anti-UTI responses [1]. TLR4 actions in bladder cells include promotion of IL-6 and IL-8 secretion by activation of MyD88-dependent or cAMP-dependent signaling pathways, and inhibition of bacterial invasion and promotion of bacterial expulsion in bladder epithelial cells [2]–[6]. In addition, neutrophil chemokine receptors, such as CXCR1, are necessary for neutrophil migration to infected sites [7]and are involved in innate immune response. CXCR1 is closely correlated with neutrophil quantity and extent of inflammatory reaction in the urinary tract.

The individual's response to UTI is variable and the susceptibility to the infection is inheritable. Lundstedt et al. found in a family study, 15% of the relatives of pyelonephritis-prone children had UTI history, whereas the value was 3% in the controls [8]. A evaluation of a familal predisposition about women with recurrent UTI described that 65.5% of mothers, 60.7% of daughters, and 48.6% of sisters of the women had a similar history [9]. Many studies discovered that genitic variations of TLR4 and CXCR1 are association with susceptibility to different type of UTIs. And TLR4(896)AG genotype and TLR4(896)G alleles could increase the risk for UTI in childhood [10], [11], CXCR1 G (2608) C gene polymorphism and expression are strong linked to acute pyelonephritis in children [12]. Reduced expression levels of CXCR1 and TLR4 in neutrophils are associated with pyelonephritis, recurrent cystitis, and asymptomatic bacteriuria in children and premenopausal women [13]–[15]. Although these studies suggest the association of gene polymorphisms and expression of TLR4 and CXCR1 to UTIs, whether the variants are associated with UTI in Chinese adults is still unknown, TLR4 and CXCR1 expression levels in different type UTIs is also not clear.

Astragalus is a Chinese herbal medicine, and Astragalus polysaccharide (APS) is its main compoents. Our previous study demonstrated that APS could induce enhancement of expression of TLR4 on bladder epithilial cells [16]. So we hope that astragalus also increases the TLR4 expression on monocytes in UTI patients.

In this study, we summarize the results of a case-control study examing whether polymorphisms and expression in TLR4 and CXCR1 are associated with susceptibility to acute pyelonephritis, chronic UTI, and acute cystitis and urethritis in UTI patients in China, and evaluating whether astragalus can boost innate immnue respons against UTI through enhancing TLR4 expression.

## Results

### 1. Clinical data

Urinary bacterial strains were identified as follows: *E. coli* in 109 patients (84.5%); *Klebsiella pneumoniae* in 5 patients (3.9%); *Proteus mirabilis* in 2 patients (1.55%); *Morganella morganii* in 2 patients (1.55%); *Enterobacter aerogenes* in 2 patients (1.55%); *Staphylococcus aureus* in 2 patients (1.55%); *Enterococcus faecalis* in 2 patients (1.55%); *Enterococcus faecium* in 3 patients (2.32%); *Monilia albicans* in 1 patient (0.78%); and *Candida glabrata* in 1 patient (0.78%).

### 2. Genotype distribution of TLR4 A (896) G and CXCR1 G (2608) C

The gene polymorphisms of TLR4 A (896) G and CXCR1G (2608) C are shown in Table 1. The genotype of TLR4 (896) AG had higher prevalence among UTI patients than in healthy controls (p = 0.03), and especially TLR4 (896) AG genotype tended to occur more frequently in acute cystitis and urethritis patients than in controls (p = 0.02). Interestingly the genotype of CXCR1 (2608) GC had lower prevalence among UTI patients than in healthy controls (p = 0.024). Compared to controls and acute cystitis and urethritis patients, the genotype of CXCR1 (2608) GC in chronic UTI patients were also lower than the two groups (p = 0.003; 0.016).

**Table 1:** Genotypic frequencies at the TLR4 A(896)G andCXCR1G(2608)C polymorphisms in patients suffering UTI compared with healthy reference populations.

Group	No.	TLR4A(896)G			CXCR1G(2608)C		
		AA	AG	GG	GG	GC	CC
healthy controls	248	227(91.5%)	21(8.5%)	0(0%)	196(79%)	50(20.2%)	2(0.8%)
UTI patients total	129	109(84.5%)	20*(15.5%)	0(0%)	111(86.1%)	15#(11.6%)	3(2.3%)
Patients with acute pyelonephritis	32	29(90.6%)	3(9.4%)	0(0%)	28(87.5%)	3(9.4%)	1(3.1%)
Patients with Chronical UTI	38	32(84.2%)	6(15.8%)	0(0%)	37(97.4%)	1†(2.6%)	0(0%)
Patients with Acute cystitis and urethritis	59	48(81.4%)	11**(18.6%)	0(0%)	46(78%)	11‡(18.6%)	2(3.4%)

*p = 0.03vs healthy controls;

**p = 0.024vs healthy controls;

# p = 0.024vs healthy controls;

† p = 0.003vs healthy controls;

‡ p = 0.016 vs chronical UTIs.

### 3.Allele frequencies of TLR4 (896) G and CXCR1 (2608) C

The prevalence of TLR4 (896) G alleles showed higher in UTI patients than that in the controls (p = 0.034), and the alleles trended to show more frequently in acute cystitis and urethritis patients than in the controls (p = 0.028). While CXCR1 (2608) C alleles had lower prevalence in chronic UTI than in the controls (p = 0.003). Compared to acute cystitis and urethritis patients, chronic UTI patients also had lower CXCR1 (2608)C allele prevalence (p = 0.003) (Table 2).

**Table 2:** Allelic frequencies at the TLR4 A(896)G andCXCR1G(2608)C polymorphisms in patients suffering UTI compared with healthy reference populations.

Group	No.	TLR4A(896)G		CXCR1G(2608)C	
		A	G	G	C
healthy controls	248	475(96%)	21(4%)	442(89%)	54(11%)
UTI patients total	129	238(92%)	20*(8%)	237(92%)	21 (8%)
Patients with acute pyelonephritis	32	61(95%)	3(5%)	59(92%)	5(8%)
Patients with Chronical UTI	38	70(92%)	6(8%)	75(99%)	1† (1%)
Patients acute cystitis and urethritis	59	107(91%)	11** (9%)	103(87%)	15‡ (13%)

*p = 0.034vs healthy controls;

**p = 0.028 vs healthy controls;

† p = 0.003 vs healthy controls;

‡ p = 0.003 vs chronical UTIs.

### 4. TLR4 expression levels in monocytes in different types of UTI

**Figure 1:**
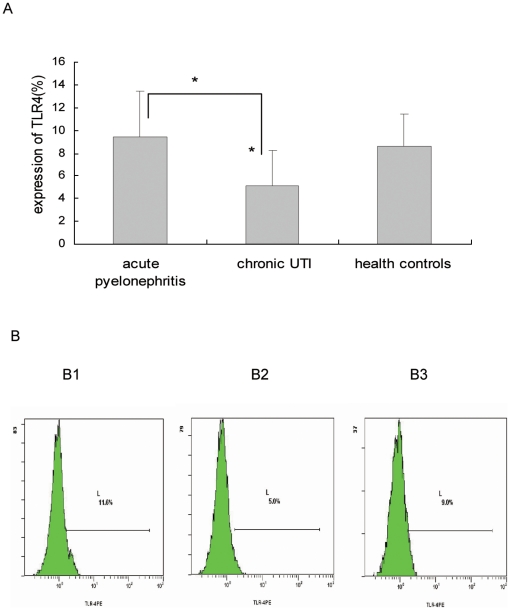
The expression levels of TLR4 on monocytes in different types of UTIs. A: Comparation of percent of TLR4 expression in different types of UTIs. B: The drawing of TLR4 detected by FCM on monocytes of UTI patients. B1: acute pyelonephritis; B2: chronical UTI; B3: health controls.

The percentage of TLR4 expression in healthy controls, chronic UTI patients, and acute pyelonephritis patients was 8.57±2.88, 5.1±3.11 and 9.4±3.1%, respectively. TLR4 expression in monocytes in patients with chronic UTI was significantly lower than in healthy controls and in patients with acute pyelonephritis (p = 0.03; 0.03) (Fig. 1).

### 5. CXCR1 expression levels in neutrophils in different types of UTI

**Figure 2:**
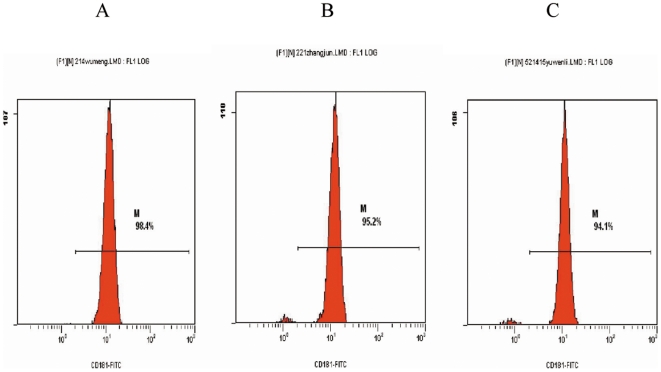
The drawing of CXCR1 detected by FCM on neutrophils of UTI patients. A:health controls; B:acute pyelonephritis; C:chronical UTI.

**Figure 3:**
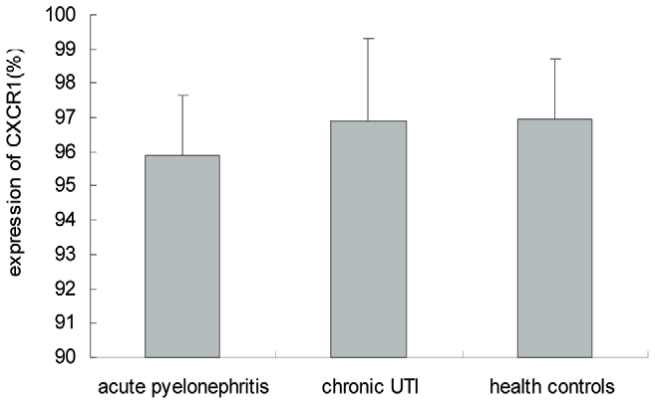
Expression of CXCR1 on monocytes of different type of UTI.

The percentage of CXCR1 expression in healthy controls, chronic UTI patients, and acute pyelonephritis patients was 96±1.75, 97±2.4 and 97±1.7%, respectively. There were no significant differences among these groups (p>0.05) (Fig. 2, Fig. 3).

### 6. TLR4 expression levels in monocytes before and after astralagus treatment

**Figure 4:**
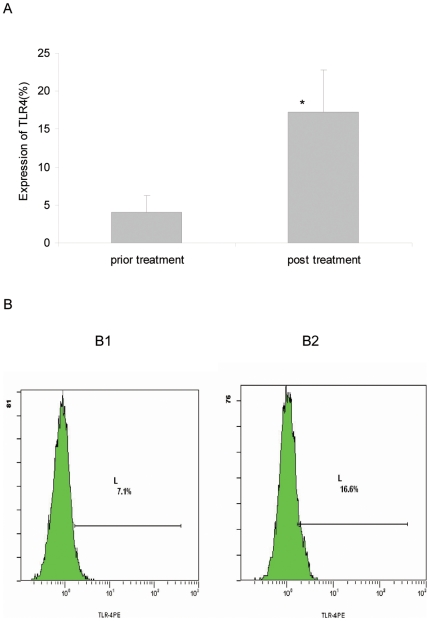
Expression of TLR4 on monocytes of pre-or post-treatment of chronic UTI. **A**: The percent of TLR4 expression on monocytes of pre-or post-treatment of chronic UTI. **B**: The drawing of TLR4 detected by FCM on monocytes of pre-or post-treatment of chronic UTI. **B1**: Pre-treatment by astralagus; B2: post-treatment by astralagus.

The percentage of TLR4 expression in chronic UTI patients before astralagus treatment (4.1±2.2%) significantly increased to 17.4±5.6% post-treatment (p = 0.001) (Fig. 4).

## Discussion

The relationship between function, expression and gene mutation of the TLR4 gene and the occurrence of UTI has interested many researchers. C3H/HeJ mice without response to LPS (TLR4 mutant mice) couldn't clear the urinary tract infection. The reason lay in that they couldn't make adequate regulation on pathogens. Maybe this defection was related to TLR4 gene mutation [17], [18], [19]Reported studies on children with urinary tract infection showed that TLR4 gene polymorphism test indicated gene carrier statuses at TLR4A(896)G allele were obviously related to recurrent urinary tract infection in children, while it had no relation with other kidney diseases [11]. Hawn etc. also carried out polymorphism test of several TLRs of adult women with 18–49 years old, and found that TLR4A (896) G polymorphism was related to UTI protection, but not related to pyelonephritis [20]. The results of this study show that the gene prevalence of TLR4 (896) AG and TLR4 (896) G alleles in UTI patients is higher than that in the control group, and the gene prevalence of TLR4 (896) AG and TLR4 (896) G alleles in patients with acute cystitis and urethritis is higher than that in the healthy control and acute pyelonephritis groups. These results indicate that TLR4A(896)G mutation is associated with increased risk of UTI in adults, especially with acute cystitis and urethritis, but is not associated with acute pyelonephritis and chronic UTI. Our data are in line with previous studies, which revealed similar occurrence of TLR4(896)AG genotype in rUTI in children [11]. But the previous also revealed that TLR4A(896)G polymorphism was associated with protection from rUTI, but not pyelonephritis in women 18–49 years old [20]. Obviously, TLR4A(896)G gene polymorphism are associated with adult susceptibility to UTIs, but this relationship could vary in different populations and disease types. Further surveys of more cases and different races are needed to make conclusive statements.

Acute pyelonephritis and asymptomatic bacteriuria (ABU) represent the two extremes of UTI respectively. Studies have shown that TLR4−/− and C3H/HeJ mice lost the function of TLR4 and could not clear effectively infection, acquired ABU status, suggesting that the absence of TLR4 signals can make an infected host appear asymptomatic [18], [21]. TLR5 and TLR11 had equal characters [22], [23]. But the extreme status was not exist almost in people. Then their susceptibility to UTI were also associated with TLR4 expression except polymorphism. Thus, the relationship between TLR4 expression level and the type of UTI has become a subject of interest. A survey of ABU children showed that compared with the healthy group, TLR4 expression level in the patient group is lower. Furthermore, TLR4 expression levels in children with primary ABU is lower than in children with secondary ABU [13]. However, there is still a lack of information on the relationship of TLR4 expression and other types of UTI. The results of this study show that TLR4 expression in patients with chronic UTI is significantly lower than that in the control and acute pyelonephritis groups, suggesting that chronic UTI can be the result of reduced TLR4 expression, thereby effectively inducing innate immune response.

TLR4 determines the efficiency of bacteria identification, but chemokine receptors are the ones that affect clearing of bacteria [24]. Defects in expression of IL-8 receptors CXCR1 and CXCR2 have been associated with many infectious diseases, particularly acute pyelonephritis. Mice lacking the CXCR1 homologue Mcxcr 2 frequently have acute pyelonephritis with bladder infection. As bacteria numbers increase, bacteremia prevalence and mortality rate increase to 50% [14]. Lundstedt et al. showed that CXCR1 expression in pyelonephritis-prone children is lower than that in the control group, and CXCR1 expression level in children with UTI is significantly lower than that in the control group [8], [14]. The results of this study show that the prevalence frequencies of CXCR1 (2608) GC and CXCR1 (2608) C in UTI patients and patients with chronic UTI are lower than those in the healthy and acute cystitis groups, but expression levels of CXCR1 in neutrophils were not different. These results indicate polymorphism of CXCR1 G(2608)C was associated with protection from UTIs in adults, especialy from chronical UTIs.

The over use of broad spectrum antibiotics has led to the emergence of antibiotic resistant bacteria. As a consequence, the treatment of UTIs especially chronic UTIs becomes a very difficult clinical problem. Modulating the powerful innate and adaptive immune systems of the urinary tract would have important therapeutic and prophylactic implications for the treatment of UTIs, particularly treatment of antibiotics alone is ineffective. Since the lining of the urinary tract is highly enrichedin TLR4 molecules, administering TLR4 specific ligands directly to the urinary tract could trigger TLR4 mediated innate immune responses thereby enhancing local reactivity and resistance to infection [24]. In present many immunomodulator were used in clinical treatment of UTI or animal experemen, such as thymosin, astragalus and ligands of TLR4. Although we have not known the mechanism, their use benefit in the urinary tract. APS is an agonist for TLR4 receptor, and it is antagonistic in a case of coexistence of LPS [25]. And we have demonstrated that APS was effective in inducing TLR4 expression and enhancing the anti-bacterial activity of bladder epithelial cells(BECs) [16]. In this study, we applicated astragalus injection and antibiotics simultaneously to treat chronic UTI patients, the TLR4 expression levels on monocytes in the patients increased after recovery. Thus we think that astragalus as an immunomodulator enhanced TLR4 expression and thereby increased the innate immune capability of patients with chronic UTI and promoted clearance of infection.

In conclusion, the TLR4A (896) G genotype was found to be associated with adult UTi, especially acute urethritis. TLR4 expression in the mononuclear cells of patients with chronic UTI was significantly reduced. Thus, TLR4 gene and expression tests can be used as auxiliary indicators for the diagnosis and treatment of UTI. With the increase of antibiotic resistance, the therapy of UTI patients can be supplemented with immunomodulators for improved results. The results of this study could provide reliable clinical evidence for the development and research of drugs targeting TLR4 for UTI treatment.

## Materials and Methods

### Patients

The study protocols were approved by Ethics committiee of Bthune International Peace Hospital Shijiazhuang City, Heibei Province, China. The different types of UTI included in this study are acute pyelonephritis (32 cases), chronic UTI (38 cases), and acute cystitis and urethritis (59 cases). Acute pyelonephritis were defined as a febrile infection (≧38.5°C) with significant bacteriuria, Creactive protein >20 mg/l and lack of symptoms of other infections. Acute cystitis and urethritis subjects were defined that patients appeared obvious irritative symptoms of bladder with bacteriuria or blood urine. Chronic UTI subjects were defined as a UTI episode within a 12-month time frame or 2 UTIs within 6 months. After obtaining their consent, blood samples were collected from 129 patients (104 women/25 men, age: 19–80y) with UTI at the Department of Nephrology, Bethune International Peace Hospital.

### Healthy controls

Health controls were enrolled and came from Bethune International Peace Hospital and verified to have no infectious disease history. There were 248 health controls (202women/46men) aged 20–70 years. After gaining their consent, blood samples were drawn for the tests.

### Samples and genotyping

Genomic DNA was extracted from whole blood samples using blood DNA extraction kit (Qiangen) according to the manufacturer's instructions. TLR4 A (896) G and CXCR1 G (2608) C genotypes were detected by RFLP-PCR as previously described [10], [26].

### Astragalus treatment of chronic UTI patients

Astragalus injection was purchased from Shanghai Fuda Pharmaceuticals Company. 20 patients with chronic UTI were selected to receive parenteral astralagus treatment. Each patient received 500 mg/kg/d astralagus intravenously for 1 or 2 weeks. At the same time they were injected antibiotics by same approachs. Antibiotics used included cefoperazone/sulbactarm(4 cases), cefotiam(6 cases), cefuroxime(4 cases), mezlocillin/sulbactarm(5 cases) and imipenem(1 case). Their TLR4 expression levels on monocytes were detected before and after treatment.

### Flow cytometry

TLR4 and CXCR1 protein expression was detected by flow cytometry. Three groups were detected: acute pyelonephritis patients, chronic UTI and health controls. There are 20 cases in each group. Monocytes and neutrophils were incubated for 30 min with PE- and FITC-labeled monoclonal antibodies against human TLR4 and CXCR1 (CD181) (BioLegend, USA), respectively. After washing with PBS, the stained cells were run on a flow cytometer (Beckman Coulter, USA). The percentage of TLR4 and CXCR1 was counted.

### Statistical analysis

Data were analyzed using SPSS13.0 software. Statistical significance was calculated by *X* ^2^ test and t-test. Significance was set at p<0.05.
